# Improving Policy for the Prevention of Falls Among Community-Dwelling Older People—A Scoping Review and Quality Assessment of International National and State Level Public Policies

**DOI:** 10.3389/ijph.2022.1604604

**Published:** 2022-06-27

**Authors:** Aleksandra H. Natora, Jennifer Oxley, Linda Barclay, Kelvin Taylor, Bruce Bolam, Terry P. Haines

**Affiliations:** ^1^ Accident Research Centre, Monash University, Clayton, VIC, Australia; ^2^ Department of Health, State Government of Victoria, Melbourne, VIC, Australia; ^3^ Department of Occupational Therapy, School of Primary and Allied Health Care, Faculty of Medicine, Nursing & Health Sciences, Monash University, Frankston, VIC, Australia; ^4^ Melbourne School of Population and Global Health, Faculty of Medicine, Dentistry and Health Sciences, University of Melbourne, Carlton, VIC, Australia; ^5^ School of Primary and Allied Health Care, Faculty of Medicine, Nursing & Health Sciences, Monash University, Frankston, VIC, Australia; ^6^ National Centre for Healthy Ageing, Faculty of Medicine, Nursing & Health Sciences, Monash University, Frankston, VIC, Australia

**Keywords:** older adults, injury prevention, falls prevention, community setting, public health policy, policy analysis

## Abstract

**Objectives:** Effective public policy to prevent falls among independent community-dwelling older adults is needed to address this global public health issue. This paper aimed to identify gaps and opportunities for improvement of future policies to increase their likelihood of success.

**Methods:** A systematic scoping review was conducted to identify policies published between 2005–2020. Policy quality was assessed using a novel framework and content criteria adapted from the World Health Organization’s guideline for Developing policies to prevent injuries and violence and the New Zealand Government’s Policy Quality Framework.

**Results:** A total of 107 articles were identified from 14 countries. Content evaluation of 25 policies revealed that only 54% of policies met the WHO criteria, and only 59% of policies met the NZ criteria. Areas for improvement included quantified objectives, prioritised interventions, budget, ministerial approval, and monitoring and evaluation.

**Conclusion:** The findings suggest deficiencies in a substantial number of policies may contribute to a disconnect between policy intent and implementation. A clear and evidence-based model falls prevention policy is warranted to enhance future government efforts to reduce the global burden of falls.

## Introduction

Falls among older adults living independently in the community are associated with thousands of fatalities and injuries around the world, and are recognised as a persistent and growing public health issue by the World Health Organization (WHO) [1, 2] and the Global Burden of Disease Study [3]. Falls are also relevant to the United Nations Sustainable Development Goals (SDGs) [4] and there are opportunities to embed and align falls prevention efforts within broader development agendas [5]. The vast majority of older adults live independently in the community and the falls-related injuries they experience are tremendously costly to the older person, their families and communities, as well as to social, health and aged care systems worldwide [1, 3, 6–12]. These costs are projected to increase in the coming decades as populations of older adults above the age of 65 years are expected to more than double from 700 million to over 1.5 billion by 2050 [13]. Falls prevention for community-dwelling older people (FPC) is achieved through implementation of evidence-based interventions and strategies [2, 14–19]. For the most part, interventions are underpinned by government public health policy.

Effective government policy is needed to achieve FPC objectives, to inform political decision making for successful implementation of interventions, and to support a systems-approach to falls prevention [1, 20–22]. The WHO highlighted promising public policy approaches in Canada, USA and Europe [1] to stimulate governments to develop effective public policies for falls prevention, especially in countries with ageing populations. Despite this innovation, its effect on the development of effective policies is unclear. Notwithstanding, there is some suggestion that falls prevention policy has not received sufficient political priority to impact on population health [23, 24], but it is unclear why. Indeed, the global burden of falls, which is now greater than transport injury, poisoning, drowning and burns combined, gave the impetus for the WHO’s latest release of the Step Safely Technical Package and its renewed call that “Now is the time to push the prevention and management of falls higher up the planning, policy, research and practice agenda…”[2] (p.vii).

Policy formulation comprises a process of discrete steps involving problem identification, agenda setting, adoption, implementation and evaluation [25, 26]. This process, often referred to as the “policy cycle” is rarely linear or sequential due to complexities of politics, policy and administration [20, 27–29]. Evidence from public health policy analysis can improve the progression of policy through the policy cycle and potentially increase policy impact on population health [30–37].

While the quality of public policies ultimately lies in their successful implementation [38–40], quality can also be inferred from the content of published policy documents [23, 41–44]. The WHO guideline for Developing policies to prevent injuries and violence [45] and several more recent frameworks provide guidance for policy content evaluation according to systematic criteria [41, 46–50], however FPC public policies appear not to have been reviewed in this way to date.

Given the importance of robust policy analysis in facilitating effective FPC, this paper aims to map and describe international public policy related to FPC and to identify gaps, strengths, weaknesses and opportunities for improvement of future FPC policy to increase the likelihood of their success.

## Methods

A two-phased approach was adopted to address the objectives of the study, described below.

### Phase 1: Exploration and Mapping of Policy Documents

This phase used a systematic scoping review methodology [51–54] to address the research question “What is the extent and nature of literature relating to international government policy for falls prevention among older adults living independently in the community from 2005 to 2020?” A protocol was developed using the Preferred Reporting Items for Systematic Reviews and Meta-Analyses (PRISMA) Scoping Review Guidelines and the PRISMA-SCR Checklist [54] to identify, select and chart the data.

#### Identification of Policy Documents

Publicly available literature on policies related to FPC published between 2005–2020 was identified in a primary search using two sources including 1) bibliographic databases and grey literature repositories, and 2) a desktop search of the websites of the World Health Organization and relevant government and health ministries (of Australia, New Zealand, UK, Europe, Canada and USA, and Asia) and Google. Bibliographic databases included MEDLINE (Ovid Interface); PUBMED (Ovid Interface); EMBASE (Ovid Interface); Cochrane Library (Ovid Interface); Global Health (Ovid Interface); SCOPUS; Web of Science; CINAHL; ProQuest (including ProQuest Policy File Index and PAIS Index); Open Access Theses and Dissertations (OATD). Grey literature repositories included Research Professional, ResearchGate, OpenGrey, GreyLit, InformIT, and SafetyLit platforms. A secondary search was conducted using a snowball method [55–57] of checking the narratives and references of articles found in the primary search and contacting key authors and experts in falls prevention for any other documents.

This search adopted a broad and inclusive definition of “public policy” that included policies authored or enacted by systems of government for the population they serve [25, 27, 58]. Public policy can include political agendas or statements of strategic plans or implementation and action plans, and “policy instruments” of laws and regulations, agreements, standards or guidelines, funded programs or services, public advocacy or education campaigns, or government networks or collaborations [27, 33]. Based on the WHO guideline [45], “public policy related to FPC” was deemed to be a publicly available document outlining the vision, goals, objectives, actions and mechanisms for government to prevent or reduce falls and fall-related injuries, deaths or their health consequences, among older adults living independently in the community setting.

An inclusive syntax of search terms was used (Supplementary Table S1 showing key concepts and keywords), with Boolean operators and truncation (Supplementary Table S2). The index year of 2005 was chosen based on the Australian 2005 National Falls Prevention Plan [59]. All study designs were included, and the search was limited to literature with English-language abstracts due to study time and resource limitations.

#### Selection of Policy Documents

Published and grey literature was selected using inclusion and exclusion eligibility criteria (Supplementary Table S3) and the COVIDENCE literature screening online tool (web-based systematic review management system). Two independent reviewers (AN and KT) systematically screened the identified literature for relevance, first by title and abstract and then by full-text. Literature identified with an English-language abstract that had non-English full-text were translated using Google Translator. Inter-rater reliability (IRR) scores were calculated. To increase consistency and inter-rater reliability between the reviewers’ screening of the literature, the first 100 articles were independently screened by two reviewers (AN and KT) and conflicts were resolved by discussion and consensus with a third reviewer (JO).

**FIGURE 1:**
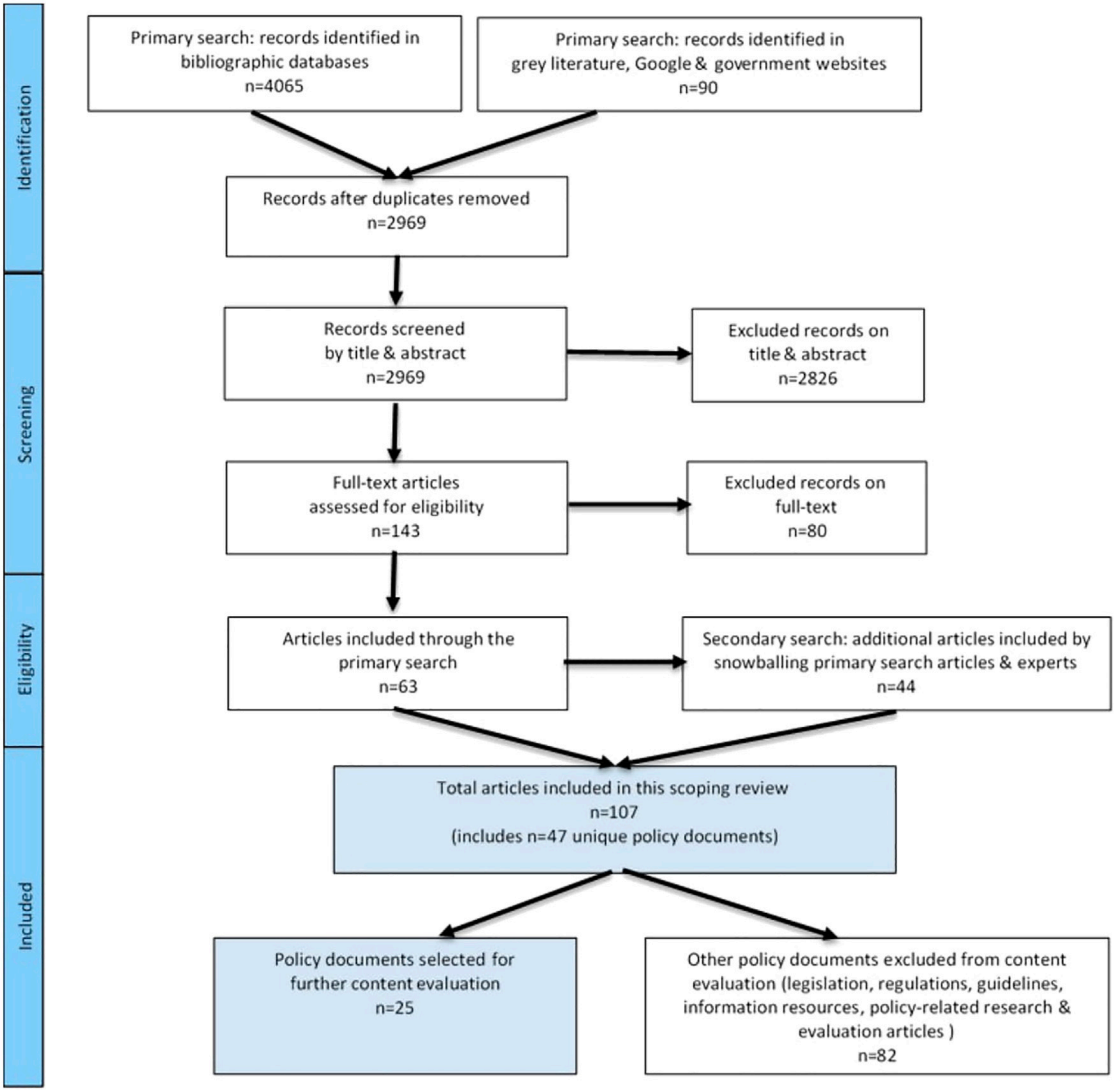
Flow diagram showing selection of public policies for this scoping review and content evaluation (Melbourne, Australia, 2020–21).

4,155 articles were identified (4,065 sourced from bibliographic databases and 90 from grey literature) (Figure 1), Following the screening and inclusion/exclusion process, the primary search for literature yielded 63 articles, and the secondary search found a further 44 articles, hence a total of 107 articles were included in the review, 47 of which were unique public policy documents.

#### Data Extraction and Assessment

Relevant data was extracted from the included 107 articles and imported into a Microsoft Excel (2019) spreadsheet. The chart was iteratively refined as data were extracted, and as secondary search articles were found. Data of interest included broad characteristics of author, year of publication, type of publication, search source, name of policy, country of origin, jurisdiction of policy, type of policy document, policy framing, and evidence of evaluation. These characteristics were collated and descriptive statistics applied.

### Phase 2: Content Evaluation of Selected Policy Documents

In addition to identifying and mapping policy documents, a content evaluation was undertaken to assess the quality of policies and address the research question “What are the strengths and weaknesses of existing FPC policies?”

#### Data Source

From the 107 identified articles, a sub-set of FPC public policies, strategic plans, action plans and position statements (*n* = 25) were selected for further content evaluation using our novel framework. Other documents such as legislation and regulations, guidelines and information resources, policy-research and evaluation articles, and those not written in English were excluded. For the national and state policies that had multiple iterations, only the most recent published policy version was included.

#### Data Extraction

There is no one internationally agreed method for policy content analysis [23, 41–44]. Hence, to assess the quality of FPC policies identified in this scoping review, a novel content evaluation framework was constructed that included 20 policy criteria adapted from two internationally recommended guidelines developed by the WHO for policy development in injury and violence prevention [45] and the Government of New Zealand (NZ) Policy Quality Framework [50] and checklist [60] that were designed to increase the quality of policy development across all government sectors.

Microsoft Excel (2019) worksheets were developed to allow two reviewers (AN and KT) to independently read the selected 25 policy documents and record categorical scores (YES = 1, NO = 0, UNCLEAR = 0.5) for the presence or absence of text in the documents that met each of the pre-defined criteria. IRR scores were generated. Discrepancies were discussed with a third reviewer (JO).

#### Data Analysis

Quantitative and qualitative analyses of policy content were undertaken. The proportion of policies that met each of the criteria were summed and expressed as percentages. Percentage scores of the two reviewers were averaged and aggregate percentages for the 20 criteria for the evaluated 25 policies were generated. In addition, reviewer observations were thematically assessed. Excel radar charts were generated to illustrate the policy relationships with the criteria.

## Results

### Overview of Policy Documents

A total of 107 articles were selected for inclusion in the review. Assessment of their broad characteristics revealed that the majority of articles originated from the USA (*n* = 47), followed by Australia (*n* = 26), and Canada (*n* = 14). Additional articles were identified from the UK (*n* = 4), New Zealand (*n* = 3), China (*n* = 3), Singapore (*n* = 1), Switzerland (*n* = 1), Netherlands (*n* = 1), European Region (*n* = 5), and included two global-oriented WHO reports. The majority of articles (72%) were descriptive reviews and research commentaries (20 from bibliographic databases, 57 from grey literature) and the remaining articles (28%) related to policy evaluations (14 from bibliographic databases and 16 from grey literature).

Further assessment of these articles identified 47 unique government policies relating to FPC, i.e., some policies were described by multiple articles. An operational definition of “policy” was provided in only four articles [21, 61–64]. These policy definitions were broadly consistent with the policy definition used in this scoping review. Only eight articles specified the guiding theoretical or conceptual framework on which the policies were based, and these included the public health approach [65–67], social and environmental determinants of health [68, 69], prevention continuum [62], quality improvement [70] and a legal framework [71].

Table 1 outlines the key characteristics of 47 policies included in this review. These policy documents represented Australia (*n* = 13), Canada (*n* = 6), China (*n* = 3), European region (*n* = 3), Netherlands (*n* = 1), New Zealand (*n* = 3), Singapore (*n* = 1), UK (England, Ireland, Scotland) (*n* = 4), and USA (*n* = 13). Of these policies, the majority (75%) were national, 19% were from state government jurisdictions, and 6% were multi-national in the European region. Policy authors included national or state government health ministries or agencies, government-funded national advisory organisations (e.g., National Council of Aging (NCOA) in the USA) or multi-agency consortia (e.g., British Columbia Fall and Injury Prevention Coalition (BCFIPC) in Canada and the Prevention of Falls Network for Dissemination (ProFouND) and EuroSafe in Europe).

**TABLE 1:** Details of 47 policy documents identified in the scoping review and their key characteristics (sorted alphabetically by Country/State), (Melbourne, Australia, 2020–21).

	Authoring agency	Year	Title of policy	Language	Country/State	Jurisdiction level	Policy Type	Policy Frame—Single Issue Falls-specific policy or Multi-issue policy that Includes falls in remit
1.	National Public Health Partnership (NPHP) [59]	2005	National Falls Prevention Plan 2004 Onwards	English	Australia	National	Strategic plan	Falls Specific
2.	National Public Health Partnership (NPHP) [102]	2005	The National Injury Prevention and Safety Promotion Plan: 2004 – 2014	English	Australia	National	Strategic plan	Multi-issue: Injury prevention
3.	Hill et al. & National Health and Medical Research Council (NHMRC) & Victorian Department of Health [103]	2011	Community Falls Prevention Sustainability Guidelines: (supporting document to the Partnership Grant: Reducing falls among older people in Victoria: better evidence, better targeting, better outcomes.)	English	Australia	National	Government guideline	Falls specific
4.	Department of Health and Ageing [104]	2011	Don’t fall for it. Falls can be prevented! A guide to preventing falls for older people	English	Australia	National	Government guideline/consumer resource	Falls specific
5.	Department of Health [105]	2020	National Injury Prevention Strategy: 2020–2030. Draft for consultation	English	Australia	National	Strategic plan	Multi-issue: Injury prevention
6.	NSW Health Department [106]	2011	Prevention of Falls and Harm from Falls among Older People 2011–2015	English	Australia, New South Wales	State	Strategic plan	Falls specific
7.	NSW Health Department [107]	2005	NSW Management Policy to reduce fall injury among older people 2003–2007	English	Australia, New South Wales	State	Strategic plan	Falls specific
8.	QLD Department of Health, Division of Chief Health Officer [108]	2009	Strategic directions for injury prevention and safety promotion 2009–2012	English	Australia, Queensland	State	Strategic plan	Multi-issue: Injury prevention
9.	Department of Health and Human Services [109]	2011	Victorian Public Health and Wellbeing Plan 2011–2015	English	Australia, Victoria	State	Strategic plan	Multi-issue: Public health
10.	Department of Health and Human Services [110]	2015	Victorian Public Health and Wellbeing Plan 2015–2019	English	Australia, Victoria	State	Strategic plan	Multi-issue: Public health
11.	Department of Health and Human Services [111]	2019	Victorian Public Health and Wellbeing Plan 2019–2023	English	Australia, Victoria	State	Strategic plan	Multi-issue: Public health
12.	WA Health Department [112]	2012	WA Health Promotion Strategic Framework 2012–2016	English	Australia, Western Australia	State	Strategic plan	Multi-issue: Health promotion
13.	WA Health Department [113]	2017	WA Health Promotion Strategic Framework 2017–2021	English	Australia, Western Australia	State	Strategic plan	Multi-issue: Health promotion
14.	Government of Canada [114]	2006	Healthy Aging in Canada: A new vision, a vital investment. A Discussion Brief prepared for the Federal, Provincial and Territorial Committee of Officials (Seniors)	English	Canada	National	Action plan	Multi-issue: Healthy Ageing
15.	Government of Canada [115]	2020	New Horizons for Seniors Program	English and French	Canada	National	Government grant program	Falls specific
16.	Public Health Agency of Canada [116]	2016	You CAN prevent falls!	English	Canada	National	Government guideline/consumer resource	Falls specific
17.	Public Health Agency of Canada [117]	2005	Report on Seniors’ falls in Canada	English	Canada	National	Government policy document	Falls specific
18.	Public Health Agency of Canada [118]	2014	Seniors’ falls in Canada. Second Report	English	Canada	National	Government policy document	Falls specific
19.	BC Injury Research and Prevention Unit and Ministry of Health, Scott et al. [119]	2014	The Next Wave of Falls Prevention in British Columbia, A special report by the BC Fall and Injury Prevention Coalition (BCFIPC)	English	Canada, British Columbia	State	Government policy document	Falls specific
20.	The State Council, The People’s Republic of China [120]	2016	National Action Plan for Disability Prevention in China 2016–2020	Abstract in English, Policy in Chinese	China	National	Action plan	Multi-issue: Public Health and Disability
21.	People’s Republic of China [121]	1996 to current	Law of the People’s Republic of China on Protection of the Rights and Interests of the Elderly	Abstract in English, Policy in Chinese	China	National	Legislation	Multi-issue: Rights of Elderly
22.	National Health and Family Planning Commission, The People’s Republic of China [122]	2016	Healthy China 2030	Abstract in English, Policy in Chinese	China	National	Action plan	Multi-issue: Public Health
23.	European Commission, The European Innovation Partnership on Active and Healthy Ageing [123]	2011	Strategic Implementation Plan for the European Innovation Partnership on Active and Healthy Ageing, Steering Group Working Document	English	Europe/Belgium	Multi-national	Implementation Plan	Multi-issue: Healthy Ageing
24.	European Commission, The European Innovation Partnership on Active and Healthy Ageing [124]	2013	Action Plan A2: Specific Action on Innovation in Support of ‘Personalized Health Management, starting with a Falls Prevention Initiative’	English	Europe/Belgium	Multi-national	Action Plan	Falls specific
25.	European Stakeholders Alliance for Active Ageing through Falls Prevention (ESA on Falls), EuroSafe Alliance & Prevention of Falls Network for Dissemination (ProFouND) [125]	2015	Active ageing through preventing falls “Falls prevention is everyone’s business”: Joint Declaration	English	Europe/Netherlands	Multi-national	Position statement	Falls specific
26.	Netherlands Ministry of Health [126]	2020	National Health Policy 2020–2024 (Landelijke-Nota-Gezondheidsbeleid-LNG-2020–2024)	Abstract in English, Policy doc in Dutch	Netherlands	National	Strategic plan	Multi-issue: Public Health
27.	New Zealand Government, Accident Compensation Corporation (ACC) [127]	2005	Preventing Injuries from Falls: The National Strategy 2005–2015	English	New Zealand	National	Strategic plan	Multi-issue: Injury
28.	New Zealand Government, Accident Compensation Corporation (ACC) [128]	2005	National Injury Prevention Strategy 2004–2014	English	New Zealand	National	Strategic plan	Multi-issue: Injury
29.	New Zealand Associate Minister of Health [129]	2016	Healthy Ageing Strategy	English	New Zealand	National	Action Plan	Multi-issue: Healthy Ageing
30.	Singapore Ministry of Health [130]	2018	I feel young in my Singapore! Action Plan for Successful Ageing	English	Singapore	National	Action Plan	Multi-issue: Healthy Ageing
31.	Public Health England [131]	2017	Falls and fracture consensus statement: Supporting commissioning for prevention (and resource pack)	English	UK, England	National	Position statement	Falls specific
32.	Health Service Executive & National Council on Ageing and Older People & Department of Health and Children [132]	2008	Strategy to Prevent Falls and Fractures in Ireland’s Ageing Population	English	UK, Ireland	National	Action Plan	Falls specific
33.	Scottish Government [133]	2014	The Prevention and Management of Falls in the Community: A Framework for Action in Scotland 2014/2016	English	UK, Scotland	National	Action Plan	Falls specific
34.	Scottish Government, Chief Nursing Officer’s Directorate [134]	2019	National Falls and Fracture Prevention Strategy 2019–2024 draft (Consultation version)	English	UK, Scotland	National	Strategic plan	Falls specific
35.	Centers of Disease Control and Prevention (CDC), National Center for Injury Prevention and Control (NCIPC) [135]	2015	A CDC Compendium of Effective Fall Interventions: What Works for Community-Dwelling Older Adults. 3rd Edition	English	USA	National	Government guideline	Falls specific
36.	Centers of Disease Control and Prevention (CDC) [136]	2015	Preventing Falls: A guide to implementing effective community-based falls prevention programs. 2nd Edition	English	USA	National	Government guideline	Falls specific
37.	Centers of Disease Control and Prevention (CDC), National Center for Injury Prevention and Control (NCIPC) [137]	2017	STEADI - Older Adult Falls Prevention: Patient & Caregiver Resources: What you can do to prevent falls	English	USA	National	Government guideline/consumer resource	Falls specific
38.	National Council On Aging (NCOA) [138]	2005	Falls Free®: Promoting a National Falls Prevention Action Plan 2005	English	USA	National	Action Plan	Falls specific
39.	National Council on Aging (NCOA), Cameron et al. [139]	2015	Falls Free®: 2015 National Falls Prevention Action Plan	English	USA	National	Action Plan	Falls specific
40.	National Council on Aging (NCOA), Beattie and Schneider [140]	2012	State Policy Toolkit for Advancing Falls Prevention	English	USA	National	Government policy document/guideline	Falls specific
41.	National Prevention Council [141]	2011	National Prevention Strategy: America’s Plan for Better Health and Wellness	English	USA	National	Strategic plan	Multi-Issue: Public Health
42.	National Prevention, Health Promotion & Public Health Council (National Prevention Council) [142]	2016	Healthy Aging in Action: Advancing the National Prevention Strategy	English	USA	National	Strategic plan	Multi-issue: Healthy Ageing
43.	United States Senate, Special Committee on Aging [143]	2019	Falls Prevention: National, State, and Local Solutions to Better Support Seniors	English	USA	National	Government policy document	Falls specific
44.	U.S. Department of Health and Human Services, Administration for Community Living (ACL) [144]	2016	Older Americans Act (OAA) 2016 Reauthorization	English	USA	National	Legislation	Multi-issue: Community Social Services
45.	U.S. Department of Health and Human Services [69]	2010	Healthy People 2020	English	USA	National	Strategic plan	Multi-issue: Public Health
46.	U.S. Department of Health and Human Services [145]	2020	Healthy People 2030	English	USA	National	Strategic plan	Multi-issue: Public Health
47.	U.S. Congress [146]	2006	H.R.5608 - Keeping Seniors Safe From Falls Act; and Report on S.1531—Keeping Seniors Safe from Falls and Reauthorization of the Traumatic Brain Injury Act	English	USA	National	Legislation	Falls specific

The policies included strategic plans and action/implementation plans, position statements, and other policy documents such as legislation, guidelines and information resources for consumers, practitioners and organisations in the community and government grant funding program. Twenty four of the policies (51%) were single-issue policies specifically for falls prevention, whereas the other 23 policies (49%) were multi-issue policies that included falls prevention in their remit. All of the multi-issue policies were from the health sector, and related to injury prevention, healthy ageing, health promotion or public health, and none were from the aged care sector.

### Content Evaluation of Selected Policy Documents

The results of our content evaluation of the selected 25 FPC policy documents are presented in Table 2. Overall proportions of the policy documents that met the 20 criteria are expressed as aggregate percentages of all policies. In addition, reviewer observations for each criteria are included. A traffic light system was applied to benchmark and highlight strengths and weaknesses of the policies. Green areas indicate well met criteria, i.e., 75 percent or more policies included the criteria (such as planned interventions, lead agency, timing and rationale). Yellow areas show the criteria that were included in 50–75 percent of the policies, and included stakeholder diversity and consumer involvement, and monitoring and evaluation. Red areas represent the criteria most deficient in the policies, namely quantified objectives, ministerial/ministry approval, allocated budget and risk and mitigation.

**TABLE 2:** Policy content evaluation framework showing aggregate results for selected 25 policy documents, (Melbourne, Australia, 2020–21).

Content Analysis Framework	Criteria (n = 20)	Criteria Description/key question used by reviewers to score the presence or absence of text in the policy document as Yes (1), No (0) or unclear (0.5)	Proportion of 25 policy documents that met the criteria (*IRR) (and traffic light colour indication 0-49% Red, 50-74% Yellow, 75-100% Green)	Reviewer Observations
World Health Organization (WHO) guideline for policy-makers and planners for developing policy to prevent injuries and violence [45]	Quantified objectives	W1 - Do objectives include reduction in falls burden by a quantified amount?	20%	Although ALL policies specified broad aims for falls prevention, only 19% policies included quantified objectives or measurable targets for reduction of injury incidence rates.
	Time frame	W2 - Is there a clear time frame for the implementation of the policy	61%	The average time frame for falls prevention specific policies was 3-4 years, compared to general policies that were for an average of 10 years.
	Target population	W3 - Is the target population clearly defined?	80%	Most policies targeted an undefined population of ‘older people’, with some explicitly defining them to be adults over the age of 60 or 65 years. One policy from Ireland targeted those above 75 years of age, and one policy from USA specified adults of 50+ years.
	Multisector involvement	W4 - Is there participation of different stakeholders in the policy formulation?	64%	Stakeholders commonly involved were from the health and aged care sectors.
	Planned interventions	W5 - Are there planned interventions that will be implemented in order to achieve the specified objectives?	77%	Specific falls prevention policies proposed a range of interventions, usually aligned with Cochrane-level evidence for community falls prevention (refs), to multi-faceted, ecological and health system approaches and strategies targeting individual older people, health professionals and community organisations that work with them, and commonly cited prominent government guidelines such as the CDC US Compendium of Effective Falls Interventions (ref).
				Broad policies that included community falls prevention in their remit centred around 3 issues: increasing falls prevention knowledge among older people, increasing physical activity opportunities for older people and enhancing falls risk management by clinicians and health professionals working in the community.
	Lead agency	W6 - Is the public administrative body that is responsible for the development and outcomes of the policy specified?	76%	In most cases the administrative body of the policy was a Health Ministry, or public health related government agency. For the other 23% of policies, policy development and implementation was implied to be a shared responsibility of diverse agencies and levels of government (e.g. NZ ACC, US NCOA, European Commission, or Europe’s ProFound network). Sometimes, responsibility for implementation was apportioned to a yet-to-be-formed lead agency as one of the aims of the policy (e.g. Australian Injury Prevention Strategy 2020-2030).
	Budget	W7 - Is a budget to finance the policy development mentioned or implicit within the policy?	12%	Few policies stated a specific budget source for the policy development or implementation. Policies with no dedicated budget for policy development, often included a call for resources to be allocated as one of the implementation actions desired by the policy.
	Monitoring and evaluation mechanism	W8 - Is there a mechanism already developed or in process for monitoring policy implementation and evaluation of its effectiveness in achieving the specified objectives?	52%	Only half of the policies nominated an established institution, method or agreed framework ready to be utilised for M&E. The nominated institution for evaluation was usually external to the lead agency responsible for developing or implementing the policy. Typically nominated were process evaluations of policy implementation programs but not evaluations of the overarching policies themselves. Few policies indicated falls-injury outcome evaluation, and two indicated intent to carry out economic evaluation of implementation programs.
	Government minister / ministry approval	W9 - Is there formal approval for policy development by government minister/ministry?	42%	Policies demonstrated formal government approval by including a forward/executive summary signed by a government official, an authorisation signature within the document.
New Zealand Government policy quality framework and checklist for developing policy across government [50, 60]	Timing	NZ1 - explains why the policy is timed now?	84%	Policy timing was usually referred to as a pressing need to tackle the ‘urgent and growing epidemiological falls burden in the community’. Some policies mentioned a government or regulatory requirement for cyclical policy review or iteration development.
	Alignment with other priorities	NZ2 - explains fit with government priorities or other policies?	63%	Falls policies often cross-referenced broader public health or preventive health policies, or policies framed as healthy and active ageing. A few policies referenced alignment with transport or road safety policies, acknowledging that many older adults fall in the community on public streets or roads and on public transport. Few policies mentioned housing policies in relation to universal design principles to ensure safe homes and buildings in which older people live.
	Rationale and evidence for government intervention	NZ3 - Does evidence support the policy? clear rationale for why government should intervene?	84%	Most falls prevention policies proposed intervention options consistent with an evidence-informed approach and the public health theoretical framework. Policies usually provided clear epidemiological rationale for why government should intervene. Most of the intervention options proposed were in keeping with an evidence-informed approach.
	Clear objectives	NZ4 - Clear policy objectives given?	79%	Most policies stated broad aims and objectives for falls prevention.
	Clear options for intervention	NZ5 - Clear options for interventions/strategies?	80%	Intervention options were usually numerous in number, variety and not prioritised and not costed.
	Target population and diversity	NZ6 - Are indigenous populations used in the analysis? Population diversity considered?	33%	When policies mentioned population diversity, it usually referred to factors of age, gender, but not cultural diversity or socio-economic diversity or functional diversity of sub-groups most at risk of falls.
	Facilitates action	NZ7 - Does it facilitate action for the decision maker?	65%	Policies usually outlined intervention actions, however few assigned specific actions to specific policy actors or stakeholders.
	Stakeholders and consumer consultation	NZ8 - Does it reflect diverse perspectives of stakeholders and public consultation?	48%	Most policies engaged diverse stakeholders in the policy development, usually limited to health and ageing sectors. Many policies were developed with a public consultation process, but few policies specifically identified consumer engagement with older people themselves.
	Risks and mitigations	NZ9 - Does it identify policy risks and mitigations?	12%	Most policies did not consider possible negative consequences of the policy, or did not identify divergent or opposing stakeholder views or how to deal with them.
	Advice for implementation	NZ10 - Does it identify clearly what needs to be implemented, by whom, when, where and why?	52%	Half the policies did not apportion specific implementation actions to specific stakeholders. Implementation or intervention actions were generally not prioritised.
	Monitoring and evaluation method	NZ11 - Is it clear how it will be monitored and evaluated?	54%	(Similar to W8) Half of the policy documents did not clearly state how monitoring or evaluation would be carried out. Some policy documents included vague intent to ‘evaluate’ or ‘review’ the policy at a later point in time, or intent to collect unspecified “evaluation data”.

Note * Inter-rater reliability (IRR) scores for the two independent reviewers performing the content evaluation were calculated using Pearson’s r Correlation Coefficient: 0.64 for the WHO criteria (strong relationship) and 0.30 for the NZ criteria (weak relationship).

**FIGURE 2:**
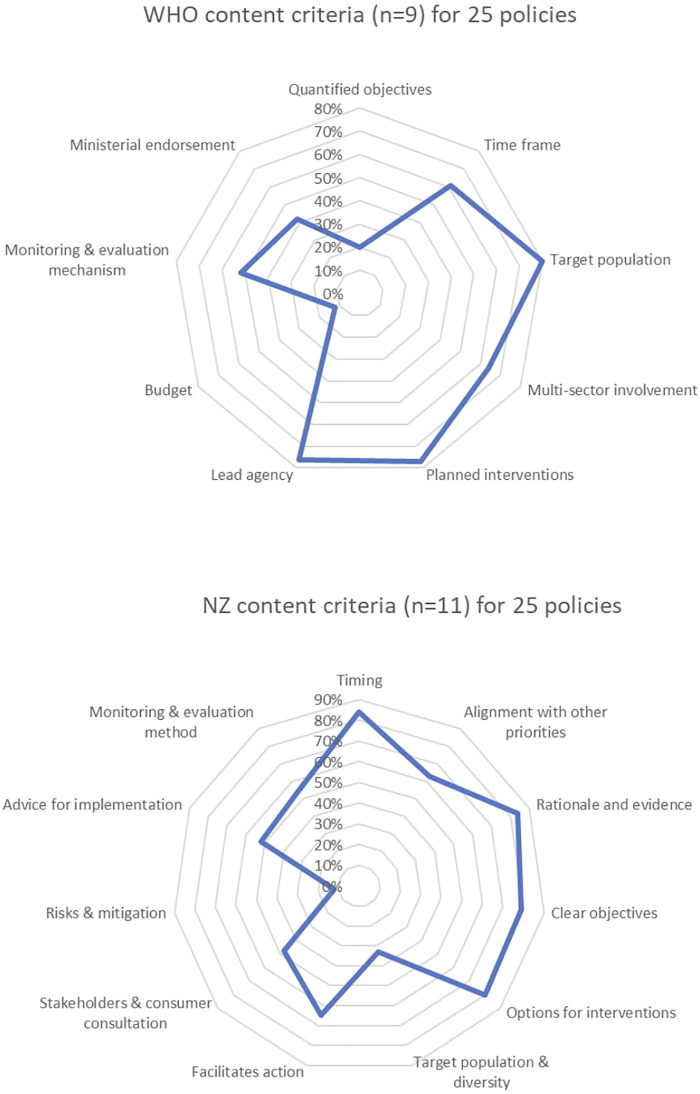
Radar charts showing percentage of public policies that met the 20 criteria of the policy content evaluation framework (Melbourne, Australia, 2020–21).

The findings of the content evaluation for both the WHO and New Zealand criteria are further visually illustrated using Excel radar charts (Figure 2). Overall, only 54% of policies met the WHO recommended 9 criteria, and only 59% of policies met the NZ recommended 11 criteria.

**FIGURE 3:**
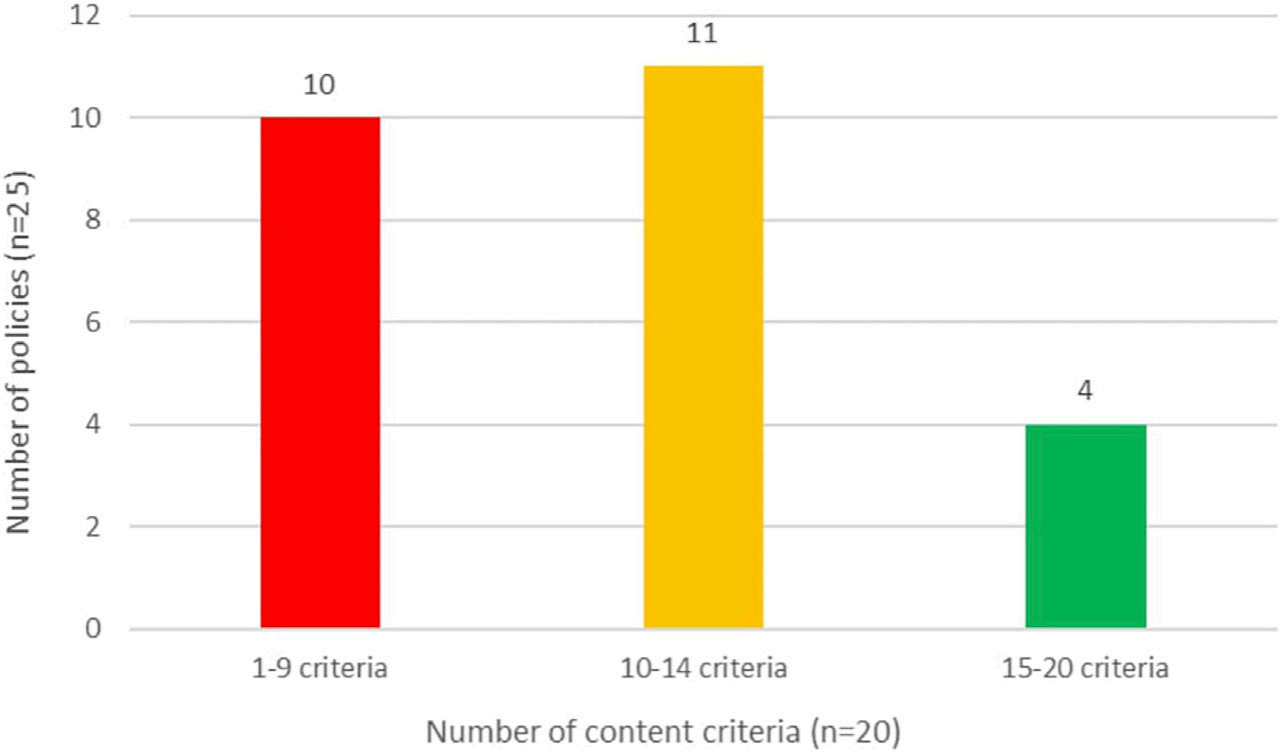
Summary of 25 public policies meeting 20 criteria in the policy content evaluation framework (Melbourne, Australia, 2020–21).

Figure 3 provides a high-level summary of the FPC policy content evaluation results. Only 4 (16%) policies met at least 75% of the criteria, while 10 (40%) met less than 50% of the criteria. It is important to note that no single policy was found to be a model policy meeting 100% of the criteria.

## Discussion

We have identified numerous policies from around the world indicating considerable effort by multiple governments to tackle falls-related morbidity and mortality. Our review provides an international policy map that has previously only been commented on for individual countries at one time [62, 63, 67, 71–73]. The documents provide a rich source of information about policy approaches, jurisdictions, stakeholders, processes and intended implementation actions that can assist policy makers in future policy development [41]. However, we identified that no single policy addressed all of the recommended policy criteria as recommended in either the WHO or NZ guidelines. There were inconsistencies in structure, duration, policy framing and other characteristics which suggest countries may be at different levels of maturity in addressing falls and falls-related injury. The heterogenous nature of the FPC policies identified also suggests individual country context factors may play a strong role in how they are designed [20]. For example, countries with national policies and corresponding sub-national/state policies (USA, Canada and Australia) may reflect their federated systems of government. This implies that administrative and non-mandatory FPC policies identified in this review have shared responsibility by multiple layers of government, making them potentially difficult to implement [38, 39].

Falls prevention in the community setting appears to have been a priority in many countries at some point, however, whether it is still a priority is unclear. We found several FPC-related policy iterations in Canada, USA, Scotland and Australia’s state of New South Wales, which may suggest these jurisdictions have had continued political attention on the issue of falls. However, some policies were expired (Australia, Ireland), which may imply that FPC may have lost status as a priority public health issue. Our evidence does not provide enough information to know the trajectory of published policies, so it is difficult to ascertain if the expired policies have ceased or simply lapsed, or been modified to take a different approach. We suggest the latter may be more likely as we found some evidence of embedding falls prevention in more recent multi-issue public health policies (NZ, Singapore, Switzerland, USA). USA and New Zealand had concurrent single-issue falls policies and embedded policies. Stand-alone falls prevention policies are by nature focused and more “niche” public health policies, and could be vulnerable to changing agendas of elected governments [27], hence embedding into other policy priorities allows for strategic alignment with more mass-appealing issues [74]. Embedded FPC policies may have better prospects of attracting political attention [75, 76] and ideally more resources for implementation. Despite the presence of these policies, previous research notes that adequate political priority has not been given to falls prevention policies [22, 23, 77, 78]. We postulate that, if political priority is to be achieved, the quality of falls prevention policy formulation needs to be improved.

Some of the common deficiencies found in policy content suggest that FPC policies may be too broad in scope, lack important detail and therefore open to misinterpretation, confusion and delay in action [27, 79]. Specifically, while all of the policies aimed to prevent falls in their communities or reduce falls-related injury and their consequences, the majority lacked quantified objectives for falls-injury incidence reduction. While over three-quarters of policies stipulated target populations of “older people” (predominantly over the age of 60 or 65 years), only one-third adequately defined their size of this population or diversity of sub-groups most at risk of falls-related injury—these are important considerations for targeting and scaling of implementation interventions [80–85]. This is supported by Ma et al. [5] who have identified opportunities for falls prevention targets to be made explicit in the SDGs to address the global burden of falls.

Only half of falls policies reported inclusion of consumer consultation during development which may imply that older people as citizens and direct beneficiaries of the policies are not always front and centre of ministerial decisions and the preventable human cost of falls is not mitigated by ministers whom have power to enable system change [86, 87]. Similarly, although over three-quarters of policies articulated comprehensive intervention options, half lacked prioritisation and even fewer provided definitive timeframes, which are necessary for workable and decisive action [29, 81].

Only 42% of the policies articulated formal approval by a government minister or ministry, and only 3 policies (12%) identified risks and mitigations of potential positive or negative consequences of the policy. We highlight that attention to funding was shown by only 12% of policies specifying or implying a budget to finance the policy, limiting the allocation of resources [88]. FPC policies, which are typically siloed health sector policies, may also be missing opportunities for a systems approach and collaborative funding from equally relevant sectors (i.e., aged care, housing and transport) to raise the political priority of FPC [2, 5].

These lost opportunities may contribute to a disconnect between policy intent and implementation [24]. It is plausible that governments intentionally publish broad “high level” policies for FPC to allow some discretion to the multi-stakeholders who implement them [27] and to allow long lead times to demonstrate outcomes. However, evidence of successful implementation of other public health policies, such as road safety and suicide prevention [89–92], suggests that falls prevention policy would benefit from setting more specific objectives. This recommendation was also highlighted by prominent injury prevention researchers, in calls for national institutions to play a greater role in “precision prevention” to reduce preventable deaths and injuries, such as from falls [93].

Many policies appear to have been designed with little capacity to be evaluated. Only half of the policies we assessed met the content criteria of having a dedicated monitoring and evaluation mechanism to evaluate implementation and effectiveness in achieving objectives in place, and outcome measures were rarely clearly specified. This may suggest that some governments do not have well-developed falls surveillance information systems in place [94–96] and/or that reliable monitoring of falls among older people in the community setting is challenging [97, 98]. It may also suggest that some policies are designed without these accountability mechanisms being put in place even when data monitoring systems are available, making it impossible to evaluate the impact of the policy [99, 100].

### Limitations

The strength of this scoping review is that it is the first study to identify and examine the content of a considerable number and variety of international policies governing FPC at national and state jurisdictional levels, hence it fills an important gap in public health policy addressing the health, safety and wellbeing of community-dwelling older people. Our retrospective content evaluation of FPC reveal how intertwined policy content is, and where there is scope to enhance the comprehensiveness of policy documents in ways that are likely to increase the impact of these policies. Future use of evidence-based policy development checklists and criteria is encouraged, particularly to strengthen policies during development and to review them when in evaluation stage.

A limitation of this scoping review is its reliance on published government policy documents in the English language, hence our findings may not be generalisable to all countries, particularly those low to middle income countries. Our search strategy made considerable effort to comprehensively and systematically identify relevant policy documents with at least an English-language abstract, and Google Translator was used for policies with non-English full text, such as policies for China and the Netherlands. Some governments may not have published their policies, or may have outsourced them to non-government organisations, so they may have been missed by the search strategy we employed. To the best of our knowledge, the identified policy documents from a large range of countries is the most comprehensive collection to date and will provide a good starting point for further research.

This review excluded government policy authored by local/provincial government jurisdictions and for falls prevention directed at non-community setting older people, i.e., those in primary/acute/hospital settings and residential aged care/nursing home settings, so it is possible that our quality assessment of this group of policies might reveal different results. The policies reviewed in this study were high-level national and state jurisdictional documents and due to the nature of their content may have lacked the details we were assessing, hence affecting the results. Although the content criteria were adapted from internationally recommended policy development guidelines they were limited and open to interpretation and our low-moderate inter-rater reliability (IRR) scores reflected the reviewers’ differing levels of knowledge and familiarity with the topics of falls prevention, public policy, as well as the guidelines. While we assessed whether recommended criteria were stated in the policy documents, it is also important to acknowledge that published policy documents might not reflect the totality of the policy process [40].

Finally, we note that although this review focused on good quality policy content, the quality of policy is ultimately determined by its effective implementation and impact [34, 41, 101]. This warrants further research to understand which of the FPC policies identified in this scoping review may have effectively led to delivery of the intended interventions for community-dwelling older people and ultimately led to a reduction in falls, fall-related injuries and other costs associated with falls.

### Conclusion

This scoping review has shown, encouragingly, that governments around the world are actively pursuing the prevention of falls in community settings by developing a variety of national and state-level public policies. Benchmarking policy content using broad internationally recommended criteria for policy development revealed several content deficiencies in FPC policies which may contribute to a disconnect between policy intent and implementation. While application of these criteria may assist policy makers to improve future falls prevention policies, there is a need for a more focused, clear and evidence-based model policy for falls prevention among community-dwelling older people to enhance future government efforts.
