# Supplementary material for: Improving Policy for the Prevention of Falls Among Community-Dwelling Older People—A Scoping Review and Quality Assessment of International National and State Level Public Policies
Source: Int J Public Health. 2022 Jun 27;67:1604604. doi: 10.3389/ijph.2022.1604604 (PMC9272743; doi:10.3389/ijph.2022.1604604)
Supplement: Supplementary file 1 [file Table1.docx]

**International Journal of Public Health**

**Review Article Title:**

Improving policy for the prevention of falls among community-dwelling older people - A scoping review and quality assessment of international national and state level public policies.

Supplementary Table S1: Literature search terms, key concepts and key words.

| Concept 1 |  | Concept 2 |  | Concept 3 |  | Concept 4 |
| --- | --- | --- | --- | --- | --- | --- |
| **Policy***  Policy Making*  Public policy  Public health  Primary health  Law  Regulat*  Plan  Framework  Strategy  Agenda  Directive  Priority  Agreement  Consensus  Statement  Memorandum  Campaign  Initiative  Standard  Guideline  Recommend*  Program*  Tax  Expenditure  Spend*  Fund*  White paper  Green paper  Consult*  Partner*  Collaborat*  Policy evaluation  Policy assessment  Policy review | AND | **Government**  Department  Ministry  Agency  National  Federal  State  jurisdiction | AND | **Accidental falls**  Accidents home  Fall*  Fall-related  Falls prevention  Accident prevention  Primary prevention  Reduction  Decrease  Control  Fall*-related injury  Injurious falls  Fatal falls | AND | **Older adult**  Older person  Elder*  Frail  Geriatric  Senior  Aged  65+ years  Middle aged  45+ years  Community-dwelling  Living at Home  Independent |
