# Supplementary material for: Improving Policy for the Prevention of Falls Among Community-Dwelling Older People—A Scoping Review and Quality Assessment of International National and State Level Public Policies
Source: Int J Public Health. 2022 Jun 27;67:1604604. doi: 10.3389/ijph.2022.1604604 (PMC9272743; doi:10.3389/ijph.2022.1604604)
Supplement: Supplementary file 2 [file Table2.docx]

**International Journal of Public Health**

**Review Article Title:** Improving policy for the prevention of falls among community-dwelling older people - A scoping review and quality assessment of international national and state level public policies.

Supplementary Table S2: Literature search strategy, example syntax for Medline/OVID, 19/09/2021.

| 1. Policy/ |
| --- |
| 2. Policy Making/ |
| 3. public policy/ or health policy/ or health care reform/ or international health regulations/ |
| 4. (policy or policies).mp. [mp=title, abstract, heading word, drug trade name, original title, device manufacturer, drug manufacturer, device trade name, keyword, floating subheading word, candidate term word] |
| 5. (law or laws or legal or legislat*).mp. [mp=title, abstract, heading word, drug trade name, original title, device manufacturer, drug manufacturer, device trade name, keyword, floating subheading word, candidate term word] |
| 6. regulat*.mp. [mp=title, abstract, heading word, drug trade name, original title, device manufacturer, drug manufacturer, device trade name, keyword, floating subheading word, candidate term word] |
| 7. (plan or framework or strategy or agenda or direct* or priorit* or agreement or consensus or statement or memorandum).mp. [mp=title, abstract, heading word, drug trade name, original title, device manufacturer, drug manufacturer, device trade name, keyword, floating subheading word, candidate term word] |
| 8. (campaign or initiative or standard or guideline or program or tax* or expenditure or spend*).mp. [mp=title, abstract, heading word, drug trade name, original title, device manufacturer, drug manufacturer, device trade name, keyword, floating subheading word, candidate term word] |
| 9. ("white paper" or "white-paper").mp. [mp=title, abstract, heading word, drug trade name, original title, device manufacturer, drug manufacturer, device trade name, keyword, floating subheading word, candidate term word] |
| 10. ("green paper" or "green-paper").mp. [mp=title, abstract, heading word, drug trade name, original title, device manufacturer, drug manufacturer, device trade name, keyword, floating subheading word, candidate term word] |
| 11. (consult* or partner* or collaborat*).mp. [mp=title, abstract, heading word, drug trade name, original title, device manufacturer, drug manufacturer, device trade name, keyword, floating subheading word, candidate term word] |
| 12. exp Public Health/ |
| 13. 1 and 12 |
| 14. Primary Health Care/ |
| 15. 1 and 14 |
| 16. exp Government/ |
| 17. Government Regulation/ |
| 18. (government or department or ministry).mp. [mp=title, abstract, heading word, drug trade name, original title, device manufacturer, drug manufacturer, device trade name, keyword, floating subheading word, candidate term word] |
| 19. ((policy* or program* or initiative) adj8 government).mp. [mp=title, abstract, heading word, drug trade name, original title, device manufacturer, drug manufacturer, device trade name, keyword, floating subheading word, candidate term word] |
| 20. ((national or state*) adj4 (policy or government)).mp. [mp=title, abstract, heading word, drug trade name, original title, device manufacturer, drug manufacturer, device trade name, keyword, floating subheading word, candidate term word] |
| 21. ((policy or policies) adj4 (impact* or evaluat* or review)).mp. [mp=title, abstract, heading word, drug trade name, original title, device manufacturer, drug manufacturer, device trade name, keyword, floating subheading word, candidate term word] |
| 22. accident prevention/ or accidental falls/ or accidents, home/ |
| 23. ((fall* or "fall-related" or "falls-related") adj3 (prevent* or reduc* or decreas* or control*)).mp. [mp=title, abstract, heading word, drug trade name, original title, device manufacturer, drug manufacturer, device trade name, keyword, floating subheading word, candidate term word] |
| 24. ("fall-related injur*" or "falls-related injur*" or "injurious fall*" or "fatal fall*").mp. [mp=title, abstract, heading word, drug trade name, original title, device manufacturer, drug manufacturer, device trade name, keyword, floating subheading word, candidate term word] |
| 25. (elderly or frail or geriatric).mp. [mp=title, abstract, heading word, drug trade name, original title, device manufacturer, drug manufacturer, device trade name, keyword, floating subheading word, candidate term word] |
| 26. (older adult* or older person*).mp. [mp=title, abstract, heading word, drug trade name, original title, device manufacturer, drug manufacturer, device trade name, keyword, floating subheading word, candidate term word] |
| 27. exp aged/ or middle aged/ |
| 28. 1 or 2 or 3 or 4 or 5 or 6 or 7 or 8 or 9 or 10 or 11 or 12 or 13 or 15 |
| 29. 16 or 17 or 18 or 19 or 20 or 21 |
| 30. 22 or 23 or 24 |
| 31. 25 or 26 or 27 |
| 32. ((elder* or frail or geriatric or senior* or "older person*" or "older adult*" or aged or "middle aged") adj4 (community or "community dwelling" or home or independen*)).mp. [mp=title, abstract, heading word, drug trade name, original title, device manufacturer, drug manufacturer, device trade name, keyword, floating subheading word, candidate term word] |
| 33. 31 or 32 |
| 34. 28 and 29 and 30 and 33 |
| 35. limit 34 to (english language and yr="2005 -Current") |
