# Supplementary material for: Improving Policy for the Prevention of Falls Among Community-Dwelling Older People—A Scoping Review and Quality Assessment of International National and State Level Public Policies
Source: Int J Public Health. 2022 Jun 27;67:1604604. doi: 10.3389/ijph.2022.1604604 (PMC9272743; doi:10.3389/ijph.2022.1604604)
Supplement: Supplementary file 3 [file Table3.docx]

**International Journal of Public Health**

**Review Article Title:** Improving policy for the prevention of falls among community-dwelling older people - A scoping review and quality assessment of international national and state level public policies.

Supplementary Table S3: Inclusion and exclusion eligibility criteria for literature screening by Title & Abstract and Full-text by two independent reviewers**.

| Inclusion criteria | Exclusion criteria |
| --- | --- |
| 1. Relates to primary prevention of falls or falls-related injury. 2. Focus on older adults (50+ years old) population living independently in the community setting (i.e. living in private home, independent-living, community-dwelling; middle aged, aged). 3. Policy authored or enacted by Government aimed at National or State-level jurisdictions (e.g. Government ministry, department, agency, or collaborative/network involving Government representation, etc.). 4. Relates to falls prevention-related government/public policy or policy instruments (e.g. plan, framework, agenda, strategy, priority, law or regulation, campaign, national or state-wide initiative, guideline, standard, consultation, collaboration, funding, etc.). 5. English Title and abstract. 6. Article published between 2005 and 2020, inclusive. | 1. Does not relate to primary prevention of falls or falls-related injury (i.e. secondary or tertiary prevention such as clinical falls risk assessment, falls-injury management, treatment or rehabilitation, clinical care of falls, falls related to patient safety, falls related to sport, transport, elder abuse, violence, animal related, occupational, etc.). 2. Does not focus on older adults living independently in the community (i.e. younger than 50 years of age, alder adults in hospital care, older people living in nursing homes or residential aged care). 3. Policy not authored or enacted by government, or government policy aimed at local/provincial government jurisdiction, or individual local communities. 4. Government policy instruments of clinical practice guidelines* for health or aged care service delivery (e.g. acute/hospital care, primary care, General Practice, Allied Health care, trauma care, etc.), or government funded clinical research trials. 5. Non-English title and abstract. 6. Article published pre-2005, or after 2020. |
| NOTE *: For the purposes of this review, we perceived clinical practice guidelines for the management and prevention of falls by clinicians in community care and clinical settings as secondary prevention, hence these were excluded from this review, however we note that these are commonly endorsed or recommended by government in many countries (e.g. **American Geriatrics** Society/British **Geriatrics** Society Clinical Practice Guidelines, US Centers for Disease Control (CDC) Stopping Elderly Accidents Deaths and Injuries (STEADI) Initiative, US Preventive Services Taskforce Recommendations, UK National Institute for Health and Care Excellence (NICE) Clinical Guidelines, Australian Commission on Safety and Quality in Health Care (ACSQHC) Best Practice Guidelines). | |
| Note ** Inter-rater reliability (IRR) scores for the two independent reviewers using the screening selection criteria were calculated at Cohen’s Kappa: 0.34804 for the Title and Abstract screening (weak relationship) and 0.40971 for the Full-text screening (moderate relationship). | |
